# Characterizing Geospatial Access and Potential Barriers for Women Presenting With Breast Concerns to Bugando Medical Center in Mwanza, Tanzania

**DOI:** 10.1200/GO.22.20000

**Published:** 2022-05-05

**Authors:** Tara Friebel-Klingner, Emma Joo, Matagoro Kirahi, Nestory Masalu, Leonard Washington, Anne Rositch

**Affiliations:** ^1^ Johns Hopkins Bloomberg School of Public Health, Baltimore, MD; ^2^ Bugando Medical Center, Mwanza, Tanzania

## Abstract

In Tanzania, high breast cancer morbidity and mortality can be attributed in part to delays due to poor access to comprehensive cancer services. Bugando Medical Center (BMC) is one of four tertiary care hospitals in Tanzania. We aimed to describe the catchment area and potential barriers to access for women presenting to BMC with breast concerns.

**METHODS:**

This prospective cohort included women presenting to BMC between September 2019 and January 2021. Patients were geocoded to residential regions and a presentation rate (PR) for each region was calculated using the number of presenting patients per at-risk female population. The main travel route from each region's referral hospital to BMC was determined using Google maps. We used descriptive statistics to depict geospatial, demographic, and clinical variables.

**RESULTS:**

The catchment area included 10.5 million women from eight regions in the Lake Zone of Tanzania. We studied 547 patients aged 18-87 (mean: 44.3), 35% (n = 196) had health insurance, and mean PR was 5.26 per 100,000 women (range: 2.01-13.86). Median distance to BMC was 121 km (IQR: 3-247) and 18% of patients likely required a ferry. As distance increased, PR tended to decrease. PRs were lower for three regions where a ferry was indicated for travel (2.01, 2.32 and 3.06). There were 151 (28%) patients diagnosed with breast cancer, with a mean breast cancer PR of 1.45 per 100,000 women (range: 0.64-3.12). The proportion of breast cancers diagnosed increased with increasing travel distance. Additionally, 51 (26%) breast cancer patients were lost to follow up without any treatment.

**CONCLUSION:**

BMC has a large geographic catchment area comprised of eight regions, all with low breast cancer PR. Our findings indicate that burdensome geographic access to cancer care, lack of health insurance, and lost to follow-up, are likely contributing to the high breast cancer mortality in Tanzania.

